# Haemorrhagic risk of transperineal ultrasound‐guided prostate biopsy performed under continued antithrombotic therapy

**DOI:** 10.1002/bco2.70260

**Published:** 2026-08-04

**Authors:** Naoki Akagi, Tatsuki Kinoshita, Motoki Fujita, Riki Obayashi, Akihiro Yamamoto, Akihiko Nagoshi, Tasuku Fujiwara, Takanari Kambe, Ryo Yamamoto, Atsushi Igarashi, Yuto Hattori, Noboru Shibasaki, Mutsushi Kawakita, Toshinari Yamasaki

**Affiliations:** ^1^ Department of Urology Kobe City Medical Center General Hospital Kobe Japan

**Keywords:** anticoagulants, antiplatelet agents, caudal anaesthesia, haemorrhagic complications, prostate biopsy

## Abstract

**Objectives:**

This study aims to evaluate the safety of transperineal prostate biopsy performed with continued antiplatelet and anticoagulant therapies under caudal anaesthesia.

**Patients and Methods:**

A total of 620 consecutive patients who underwent transperineal ultrasound‐guided prostate biopsy at a tertiary care centre in Japan from March 2023 to June 2025 were retrospectively analysed. Antithrombotic agents were continued according to institutional policy, and all procedures were performed under caudal anaesthesia. Patients were categorised into a no‐antithrombotic therapy group (NA group, *n* = 474) and an antithrombotic therapy group (AP/AC group, *n* = 146). The primary endpoint was any Clavien–Dindo grade I or higher haemorrhagic complications. Univariate and multivariate logistic regression analyses were conducted.

**Results:**

Haemorrhagic events occurred in 8.9% and 11.7% of patients in the NA and AP/AC groups, respectively (*p* = 0.33). Clinically significant haemorrhage (Clavien–Dindo grade II or higher) was rare (1.1% vs. 2.1%), and only one grade III event occurred in the AP/AC group. None of the patients required blood transfusion, and no sacral hematomas, neurological deficits or thromboembolic events were observed. Multivariate analysis adjusted for prostate‐specific antigen level, platelet count, prostate volume and number of biopsy cores revealed that antithrombotic therapy was not independently associated with haemorrhagic complications (odds ratio: 1.14; 95% confidence interval: 0.60–2.08).

**Conclusion:**

Continuation of antiplatelet or anticoagulant therapy during transperineal prostate biopsy under caudal anaesthesia was not associated with a clear increase in clinically relevant haemorrhagic complications, and no anaesthesia‐related complications were observed.

## 1 INTRODUCTION

Transperineal prostate biopsy is associated with lower risks of infectious complications and rectal bleeding and has consequently been increasingly adopted as an alternative to the transrectal approach. The PREVENT randomised controlled trial further strengthened the evidence supporting the safety and diagnostic efficacy of the transperineal approach.^1^ Additionally, a recent systematic review showed an association between transperineal biopsy and low infectious complication rates while maintaining an acceptable overall safety profile.^2^

Although generally mild and self‐limiting, haemorrhagic events such as haematuria, perineal hematoma and clot retention may occur after prostate biopsy. With progressive population ageing, urologists increasingly perform prostate biopsy in patients receiving antiplatelet or anticoagulant therapy, underscoring the clinical importance of periprocedural antithrombotic therapy management.

Periprocedural management poses a clinical dilemma in patients receiving antithrombotic therapy. Discontinuation of anticoagulant or antiplatelet therapy may decrease the risk of bleeding but increase the risk of thromboembolic events, whereas continuation of anticoagulant or antiplatelet therapy raises concerns regarding bleeding complications. Previous findings from several studies, including data from our institution, indicated that procedures such as holmium laser enucleation of the prostate, robot‐assisted radical prostatectomy, and laparoscopic nephrectomy and nephroureterectomy could be performed without evidently increasing the perioperative risk of bleeding among patients continuing to receive antithrombotic therapy.^3^, ^4^, ^5^ Based on this experience, continuation strategies have been extended to prostate biopsy. Nonetheless, evidence specifically addressing the safety of continued antithrombotic therapy during transperineal prostate biopsy, particularly under neuraxial anaesthesia, remains limited.

The present study aimed to evaluate the safety of transperineal prostate biopsy performed with continued antithrombotic therapy, with all procedures performed under caudal anaesthesia.

## 2 PATIENTS AND METHODS

This single‐centre retrospective study analysed a prospectively managed cohort of 620 consecutive patients who underwent transperineal ultrasound‐guided prostate biopsy at a tertiary care centre in Japan from March 2023 to June 2025. The management of all patients receiving antiplatelet or anticoagulant therapy adhered to an institutional policy of peri‐biopsy continuation of antiplatelet and anticoagulant agents. All procedures were performed on an outpatient basis.

Caudal (sacral) epidural anaesthesia was induced by injecting 10 mL of 2% mepivacaine hydrochloride using a 22‐gauge needle. In this cohort, all procedures were performed under caudal anaesthesia.

Transperineal biopsy was performed under ultrasound guidance using an 18‐gauge core biopsy needle. Systematic double‐sextant (12‐core) biopsy was conducted in all patients according to institutional protocol. Four additional lateral peripheral zone cores were obtained in patients with a prostate volume of ≥50 mL, resulting in a maximum of 16 cores. In patients with suspicious lesions identified on prebiopsy multiparametric magnetic resonance imaging (MRI), additional targeted biopsy under ultrasound guidance was performed, and two targeted cores were acquired for each MRI‐identified lesion.

The patients were categorised into two groups according to antithrombotic therapy status at the time of biopsy: the no‐antithrombotic therapy group (NA group, *n* = 474) and the antithrombotic therapy group (AP/AC group, *n* = 146). The AP/AC group comprised patients who received antiplatelet (AP group, *n* = 100) or anticoagulant (AC group, *n* = 46) agents. Subgroup analyses of antiplatelet and anticoagulant regimens were conducted descriptively.

All complications, particularly haemorrhagic complications, such as haematuria, perineal hematoma and clot retention, were assessed during outpatient follow‐up visits at 1–2 weeks after biopsy and were classified according to the Clavien–Dindo grading system.^6^ Grade I haemorrhagic complications were defined as self‐limited haematuria or perineal hematoma requiring observation alone without additional intervention. Grade II complications included haemorrhagic events requiring active medical management, such as pharmacological treatment including transfusion, urethral catheterisation or bladder irrigation. Grade III complications were defined as haemorrhagic events requiring surgical or endoscopic intervention, including transurethral coagulation for haemostasis. The primary endpoint was the occurrence of any Clavien–Dindo grade I or higher haemorrhagic complications.

Potential factors associated with haemorrhagic complications were identified via univariate analyses conducted with the following baseline variables: age, body mass index (BMI), prostate‐specific antigen (PSA), platelet count, activated partial thromboplastin time (APTT), prothrombin time–international normalised ratio (PT‐INR), antithrombotic therapy status (NA vs. AP/AC), prostate volume and number of biopsy cores. Continuous variables were summarised as medians with interquartile ranges and were compared using the Wilcoxon rank‐sum test. Categorical variables were compared using Fisher's exact test.

Independent predictors of haemorrhagic complications were identified through a multivariate logistic regression analysis. Variables showing significant associations with haemorrhagic events in the univariate analyses (PSA level, platelet count, prostate volume and number of biopsy cores) were entered into the model along with antithrombotic therapy status (NA vs. AP/AC), which was treated as the main explanatory variable.

Statistical analyses were performed using EZR (Saitama Medical Center, Jichi Medical University, Saitama, Japan), a graphical user interface for R (The R Foundation for Statistical Computing, Vienna, Austria),^7^ with statistical significance set at a two‐tailed *p*‐value of <0.05.

This study was conducted in accordance with the Declaration of Helsinki, as revised in 2013, and was approved by the Ethics Committee of Kobe City Medical Center General Hospital (approval no. zn251015). Informed consent was obtained using an opt‐out method on the hospital website. The patients who did not opt out were included in the study.

## 3 RESULTS

A total of 620 patients were analysed, among them 474 (76.5%) were assigned to the NA group, 100 (16.1%) to the AP group and 46 (7.4%) to the AC group (Table 1). Of 100 patients in the AP group, 66 were receiving aspirin monotherapy, 26 were receiving P2Y12 inhibitor monotherapy, five were receiving aspirin plus P2Y12 inhibitor combination therapy and three were taking cilostazol. In the AC group, nine patients were treated with warfarin alone, 30 with direct oral anticoagulants (DOACs) and seven with anticoagulant plus antiplatelet combination therapy (Table 1).

**TABLE 1:** Medication regimen.

	NA group (*n* = 474)	AP group (*n* = 100)	AC group (*n* = 46)
Aspirin, *n* (%)	‐	66 (66.0)	‐
P2Y12‐I, *n* (%)	‐	26 (26.0)	‐
Aspirin + P2Y12‐I, *n* (%)	‐	5 (5.0)	‐
Cilostazol, *n* (%)	‐	3 (3.0)	‐
Warfarin, *n* (%)	‐	‐	9 (19.6)
DOACs, *n* (%)	‐	‐	30 (65.2)
Anticoagulant plus antiplatelet combination therapy^a^, *n* (%)	‐	‐	7 (15.2)

Abbreviations: AC, anticoagulant therapy; AP, antiplatelet therapy; DOACs, direct oral anticoagulants; NA, no antithrombotic therapy.

^a^ Anticoagulant plus antiplatelet combination therapy group (*n* = 7): warfarin + aspirin (*n* = 4), warfarin + clopidogrel (*n* = 2), DOACs + aspirin (*n* = 1).

The baseline characteristics of patients are summarised in Table 2. The patients in the AP/AC group (*n* = 146) were significantly older than those in the NA group (median: 76.0 vs. 72.0 years, *p* < 0.05) and showed a significantly lower platelet count (median: 19.7 × 10^9^/L vs. 21.8 × 10^9^/L, *p* < 0.05). Coagulation‐related parameters such as PT‐INR and APTT differed significantly between the NA and AP/AC groups, whereas BMI, PSA level, prostate volume and the number of biopsy cores did not.

**TABLE 2:** Patient characteristics.

	NA group (*n* = 474)	AP/AC group (*n* = 146)	*p*‐value
Age (years), median (IQR)	72.0 (66.0,76.0)	76.0 (71.0,79.7)	<0.05
BMI (kg/m^2^), median (IQR)	23.3 (21.4,25.1)	23.7 (21.5,25.6)	0.28
Platelet count (×10^9^/L), median (IQR)	21.8 (18.8,24.9)	19.7 (16.5,23.1)	<0.05
PT‐INR, median (IQR)	0.96 (0.92,1.00)	1.01 (0.96,1.17)	<0.05
APTT (s), median (IQR)	26.3 (25.9,28.8)	28.5 (26.2,30.2)	<0.05
PSA (ng/mL), median (IQR)	9.2 (6.4–15.8)	10.3 (6.2,19.9)	0.22
Prostate volume (mL), median (IQR)	38.0 (29.0,52.8)	40.0 (29.2,58.0)	0.27
Number of biopsy cores, median (IQR)	14.0 (14.0,16.0)	14.0 (14.0,17.5)	0.22
Ischaemic heart disease, *n* (%)	0 (0)	59 (40.4)	‐
Cerebrovascular disease, *n* (%)	0 (0)	36 (24.6)	‐
Carotid artery stenosis, *n* (%)	0 (0)	5 (3.4)	‐
Atrial fibrillation, *n* (%)	0 (0)	35 (24.0)	‐
Deep vein thrombosis, *n* (%)	0 (0)	4 (2.7)	‐
History of prosthetic heart valve replacement, *n* (%)	0 (0)	6 (4.1)	‐

Abbreviations: AP/AC, antithrombotic therapy group; APTT, activated partial thromboplastin time; BMI, body mass index; IQR, interquartile range; NA, no‐antithrombotic therapy; PSA, prostate‐specific antigen; PT‐INR, prothrombin time–international normalised ratio.

Postbiopsy complication rates are presented in Table 3. Haemorrhagic events occurred in 42 patients (8.9%) in the NA group and 17 patients (11.7%) in the AP/AC group (*p* = 0.33). Clinically significant haemorrhage (Clavien–Dindo grade II or higher) was detected in five patients (1.1%) in the NA group and three patients (2.1%) in the AP/AC group. A single grade III haemorrhagic event was noted in the AP/AC group; conversely, no grade III event was observed in the NA group. None of the patients required blood transfusion; however, 1.1% and 2.1% of patients in the NA and AP/AC groups, respectively, were hospitalised for haemorrhage (*p* = 0.40).

**TABLE 3:** Complications.

	NA group (*n* = 474)	AP/AC group (*n* = 146)	*p*‐value
Haemorrhage (total), *n* (%)	42 (8.9)	17 (11.7)	0.33
Clavien–Dindo grade I, *n* (%)	37 (7.8)	14 (9.6)	0.49
Clavien–Dindo grade II, *n* (%)	5 (1.1)	2 (1.4)	0.67
Clavien–Dindo grade III, *n* (%)	0 (0.0)	1 (0.7)^a^	0.24
Transfusion, *n* (%)	0 (0.0)	0 (0.0)	‐
Hospitalisation for haemorrhage, *n* (%)	5 (1.1)	3(2.1)	0.4
Sacral hematomas or nerve symptoms, *n* (%)	0 (0.0)	0 (0.0)	‐
Thrombus, *n* (%)	0 (0.0)	0 (0.0)	‐

Abbreviations: AP/AC, antithrombotic therapy group; NA, no‐antithrombotic therapy.

^a^ Transurethral coagulation.

All procedures were performed under caudal anaesthesia. No sacral hematomas or neurological deficits indicative of neuraxial bleeding were observed. Additionally, no thromboembolic events occurred during the periprocedural period.

Detailed complication profiles are presented according to specific antiplatelet and anticoagulant regimens in Tables 4 and 5. In the AP group, haemorrhagic events occurred in nine patients (13.6%) receiving aspirin monotherapy, two patients (7.6%) receiving P2Y12 inhibitor monotherapy, and one patient (33.3%) treated with cilostazol (overall *p* = 0.49); none of the patients receiving aspirin plus P2Y12 inhibitor combination therapy developed haemorrhagic complications. One patient receiving P2Y12 inhibitor monotherapy had a grade II haemorrhage. A single grade III haemorrhagic event requiring transurethral coagulation for haemostasis occurred in a patient receiving aspirin monotherapy, who recovered uneventfully without long‐term sequelae.

**TABLE 4:** Complications in the AP group.

	Aspirin group (*n* = 66)	P2Y12‐I group (*n* = 26)	P2Y12‐I + aspirin group (*n* = 5)	Cilostazol group (*n* = 3)	*p*‐value
Haemorrhage (total), *n* (%)	9 (13.6)	2 (7.6)	0 (0.0)	1 (33.3)	0.49
Clavien–Dindo grade 0, *n* (%)	57 (86.4)	24 (92.3)	5 (100.0)	2 (66.7)	0.49
Clavien–Dindo grade I, *n* (%)	8 (12.1)	1 (3.8)	0 (0.0)	1 (33.3)	0.3
Clavien–Dindo grade II, *n* (%)	0 (0.0)	1 (3.8)	0 (0.0)	0 (0.0)	0.34
Clavien–Dindo grade III, *n* (%)	1 (1.5)^a^	0 (0.0)	0 (0.0)	0 (0.0)	1

Abbreviation: AP, antiplatelet therapy.

^a^ Transurethral coagulation.

**TABLE 5:** Complications in the AC group.

	Warfarin group (*n* = 9)	DOAC group (*n* = 30)	AC + AP group (*n* = 7)	*p*‐value
Haemorrhage (total), *n* (%)	1 (11.1)	3 (10.0)	1 (14.3)	1
Clavien–Dindo grade 0, *n* (%)	8 (88.9)	27 (90.0)	6 (85.7)	1
Clavien–Dindo grade I, *n* (%)	0 (0.0)	3 (10.0)	1 (14.3)	0.78
Clavien–Dindo grade II, *n* (%)	1 (11.1)	0 (0.0)	0 (0.0)	0.35
Clavien–Dindo grade III, *n* (%)	0 (0.0)	0 (0.0)	0 (0.0)	1

Abbreviations: AC, anticoagulant therapy; AC + AP: anticoagulant plus antiplatelet combination therapy; DOAC, direct oral anticoagulant.

In the AC group, haemorrhagic events were identified in one patient (11.1%) treated with warfarin, three patients (10.0%) treated with DOACs and one patient (14.3%) receiving anticoagulant plus antiplatelet combination therapy, with no significant differences among regimens (*p* = 1.0). No grade III haemorrhagic event occurred in the AC group.

The results of univariate and multivariate analyses for factors associated with Clavien–Dindo grade I or higher haemorrhagic events are presented in Table 6. Univariate analyses showed that higher PSA level (*p* = 0.04), larger prostate volume (*p* = 0.02), greater number of biopsy cores (*p* = 0.01) and lower platelet count (*p* = 0.01) were associated with haemorrhagic events. The use of antithrombotic therapy was not significantly associated with bleeding (*p* = 0.33).

**TABLE 6:** Univariate and multivariate analyses of factors associated with any haemorrhagic complications (grade I–III vs. grade 0).

	No haemorrhage (*n* = 561)	Grade I–III haemorrhage (*n* = 59)	Univariate *p*‐value	OR	CI	Multivariate *p*‐value
Age (years), median (IQR)	73.0 (67.0,77.0)	75.0 (67.0,78.5)	0.06			
BMI (kg/m^2^), median (IQR)	23.3 (21.4,25.2)	23.9 (22.1,25.6)	0.27			
PSA (ng/mL), median (IQR)	9.1 (6.2,16.0)	10.7 (7.7,19.1)	0.04	1.00	(1.00–1.00)	0.817
Platelet count (×10^9^/L), median (IQR)	21.4 (18.5,24.7)	20.2 (16.9,22.4)	0.01	0.94	(0.88–0.99)	0.025
APTT (s), median (IQR)	26.9 (25.7,29.5)	27.3 (25.9,29.8)	0.45			
PT‐INR, median (IQR)	0.97 (0.92–1.02)	0.97 (0.94–1.04)	0.52			
NA vs. AP/AC						
NA, *n* (%)	432 (77.0)	42 (71.2)	0.33	1.14	(0.60–2.08)	0.689
AP/AC, *n* (%)	129 (23.0)	17 (28.8)				
Prostate volume (mL), median (IQR)	38.0 (29.0–53.0)	49.0 (29.2–69.0)	0.02	1.01	(1.00–1.02)	0.068
Number of biopsy cores, median (IQR)	14.0 (14.0–16.0)	16.0 (14.0–18.0)	0.01	1.02	(0.93–1.11)	0.598

Abbreviations: AP/AC, antithrombotic therapy group; APTT, activated partial thromboplastin time; CI, confidence interval; IQR, interquartile range; NA, no‐antithrombotic therapy; OR, odds ratio; PT‐INR, prothrombin time–international normalised ratio.

Multivariate logistic regression analysis revealed that antithrombotic therapy (AP/AC vs. NA) was not independently associated with haemorrhagic events (odds ratio [OR]: 1.14, 95% confidence interval [CI]: 0.60–2.08, *p* = 0.689). Lower platelet count remained significantly associated with haemorrhagic complications (OR: 0.94 per 1 × 10^9^/L increase, 95% CI: 0.88–0.99, *p* = 0.025), whereas PSA level and prostate volume were not significant after adjustment.

## 4 DISCUSSION

The findings of the present study suggest that transperineal prostate biopsy performed under continued antithrombotic therapy, including both antiplatelet and anticoagulant regimens, can be performed without a clear increase in haemorrhagic complications. In this study, the patients receiving and not receiving antithrombotic therapy showed comparable overall rates of Clavien–Dindo grade I or higher haemorrhagic events, and clinically significant haemorrhage was uncommon. During the periprocedural period, none of the patients required transfusion or developed thromboembolic events. These findings align with the results of previous studies reporting that continuation of antiplatelet therapy did not substantially increase the risk of bleeding during prostate biopsy.^8^, ^9^ Furthermore, Saito et al. and Asano et al. similarly demonstrated that continuation of antithrombotic therapy, including dual therapy, did not significantly increase the risk of serious bleeding complications in the transperineal setting.^10^, ^11^

Univariate analyses revealed that larger prostate volume, greater number of biopsy cores, higher PSA level and lower platelet count were associated with haemorrhagic events, whereas the use of antithrombotic therapy was not. Multivariate analysis adjusted for PSA level, platelet count, prostate volume and number of biopsy cores indicated that antithrombotic therapy was not independently associated with haemorrhagic complications. Although the CIs were wide owing to the limited number of events, no clear signal of an increased bleeding risk attributable to continued antithrombotic therapy was observed. Importantly, haemorrhagic events were infrequent across a broad spectrum of antithrombotic regimens, including P2Y12 inhibitors, warfarin, DOACs and combination therapy, with no significant differences found among specific regimens. The absence of an obvious excess bleeding signal with DOACs or dual therapy provides additional real‐world reassurance.^10^, ^11^, ^12^ In contrast, heparin bridging during prostate biopsy is associated with an increased risk of bleeding.^13^

An additional clinically important observation is that no thromboembolic events occurred in any group during the periprocedural period. Previous studies reported a non‐negligible risk of arterial or venous thromboembolism after perioperative interruption of antiplatelet or anticoagulant therapy, particularly in patients with drug‐eluting coronary stents or other high‐risk cardiovascular conditions.^14^, ^15^, ^16^ In our cohort of 146 patients receiving antithrombotic therapy, maintaining treatment throughout the peri‐biopsy period may have helped in avoiding a small but clinically meaningful number of thromboembolic events. This potential benefit should be weighed against any modest increase in largely manageable bleeding complications.

Another clinically relevant observation is the safety of caudal anaesthesia in this setting. Regional anaesthesia guidelines recommend caution when performing neuraxial procedures in patients receiving antithrombotic therapy.^17^, ^18^ In this series, no sacral hematomas or neurological deficits were detected. Simon et al. suggested that caudal epidural procedures could be performed safely without interrupting antithrombotic therapy.^19^ Anatomical features of the sacral canal, such as fewer venous plexuses and greater epidural fat, may contribute to a lower risk of epidural hematoma. Consistent with these observations, no neurological deficits or symptoms suggestive of sacral hematoma were observed in our cohort, providing additional reassurance regarding the safety of sacral anaesthesia under careful monitoring. Although the present findings support the feasibility of a continuation strategy, they do not resolve whether brief interruption protocols may represent an alternative approach in selected patients. Given the rarity of major haemorrhagic and thromboembolic events, future studies comparing continuation and short‐interruption strategies would likely require very large cohorts.

This study has some limitations. First, this study was retrospective in design and was conducted at a single centre, limiting the generalisability of findings. Second, minor bleeding events might have been underreported because complications were primarily assessed during follow‐up visits. Third, the number of clinically significant haemorrhagic events was relatively small, limiting statistical power and resulting in wide CIs. Fourth, the biopsy protocol used in this cohort may differ from contemporary practice patterns in some institutions. In the present study, two targeted cores were obtained per MRI‐visible lesion in addition to systematic biopsy cores, whereas some centres routinely perform more extensive targeted or saturation biopsy protocols. More extensive sampling strategies, particularly for lesions located near the urethra or neurovascular bundles, may potentially increase haemorrhagic risk. Therefore, the present findings may not be fully generalisable to all contemporary biopsy protocols. In addition, a substantial proportion of patients in the antithrombotic group were receiving aspirin monotherapy, whereas the numbers of patients receiving dual antiplatelet therapy or anticoagulant‐based combination regimens were relatively small. Therefore, caution is warranted when extrapolating these findings to less common antithrombotic regimens. Finally, subgroup analyses according to specific antithrombotic regimens were based on very few events and should therefore be interpreted as exploratory and descriptive.

## 5 CONCLUSIONS

Continuation of antiplatelet or anticoagulant therapy was not associated with a clear increase in clinically relevant haemorrhagic or anaesthesia‐related complications in this retrospective cohort of patients undergoing transperineal prostate biopsy under caudal anaesthesia; additionally, no thromboembolic events were observed. Although the limited number of major events precludes definitive conclusions, these findings support an individualised peri‐biopsy management strategy that avoids routine discontinuation of antithrombotic therapy in appropriately selected patients.

## AUTHOR CONTRIBUTIONS

**Naoki Akagi:** Conceptualisation, Data curation, Formal analysis and Writing – original draft. **Tatsuki Kinoshita:** Data curation and Investigation. **All authors:** Manuscript review and approval of the final manuscript.

## CONFLICT OF INTEREST STATEMENT

The authors declare no conflicts of interest.

## Data Availability

The data that support the findings of this study are available from the corresponding author upon reasonable request.
